# Healthcare Utilization (HCU) Reduction with High-Frequency (10 kHz) Spinal Cord Stimulation (SCS) Therapy

**DOI:** 10.3390/healthcare12070745

**Published:** 2024-03-29

**Authors:** Vinicius Tieppo Francio, Logan Leavitt, John Alm, Daniel Mok, Byung-jo Victor Yoon, Niaman Nazir, Christopher M. Lam, Usman Latif, Timothy Sowder, Edward Braun, Andrew Sack, Talal W. Khan, Dawood Sayed

**Affiliations:** 1 Department of Anesthesiology and Pain Medicine, The University of Kansas Medical Center, Kansas City, KS 66160, USA; 2 Department of Physical Medicine and Rehabilitation, The University of Kansas Medical Center, Kansas City, KS 66160, USA; 3 Department of Population Health, The University of Kansas Medical Center, Kansas City, KS 66160, USA

**Keywords:** spinal cord stimulation, chronic low back pain, healthcare utilization, cost-effectiveness, opioid use, emergency departments, outpatient visits

## Abstract

Spinal cord stimulation (SCS) is a well-established treatment for patients with chronic pain. With increasing healthcare costs, it is important to determine the benefits of SCS in healthcare utilization (HCU). This retrospective, single-center observational study involved 160 subjects who underwent implantation of a high-frequency (10 kHz) SCS device. We focused on assessing trends in HCU by measuring opioid consumption in morphine milligram equivalents (MME), as well as monitoring emergency department (ED) and office visits for interventional pain procedures during the 12-month period preceding and following the SCS implant. Our results revealed a statistically significant reduction in HCU in all domains assessed. The mean MME was 51.05 and 26.52 pre- and post-implant, respectively. There was a 24.53 MME overall decrease and a mean of 78.2% statistically significant dose reduction (*p* < 0.0001). Of these, 91.5% reached a minimally clinically important difference (MCID) in opioid reduction. Similarly, we found a statistically significant (*p* < 0.01) decrease in ED visits, with a mean of 0.12 pre- and 0.03 post-implant, and a decrease in office visits for interventional pain procedures from a 1.39 pre- to 0.28 post-10 kHz SCS implant, representing a 1.11 statistically significant (*p* < 0.0001) mean reduction. Our study reports the largest cohort of real-world data published to date analyzing HCU trends with 10 kHz SCS for multiple pain etiologies. Furthermore, this is the first and only study evaluating HCU trends with 10 kHz SCS by assessing opioid use, ED visits, and outpatient visits for interventional pain procedures collectively. Preceding studies have individually investigated these outcomes, consistently yielding positive results comparable to our findings.

## 1. Introduction

Chronic low back pain (CLBP) affects over 50 million American adults, and it is estimated that up to 85% of people are likely to experience back pain at some point in their lives [1,2,3]. In the United States alone, CLBP has an estimated indirect healthcare burden as high as USD 624.8 billion and is the leading cause of disability [4,5,6]. It is expected that both the total disability burden and disease-related healthcare costs associated with CLBP will continue to rise in the upcoming decades [5]. Healthcare utilization (HCU) among patients with CLBP includes various direct costs, such as outpatient clinic visits, emergency department visits, medications, spinal injections, and spine surgeries [4]. Additionally, there can also be significant indirect costs, as patients with CLBP often have significant comorbidities, such as mental health conditions, metabolic disorders, or sleep disorders [7,8]. It has been estimated that within the first year of a diagnosis of CLBP, the median total healthcare cost can be over USD 6000 [4]. High healthcare utilizers, delineated as individuals positioned within the upper quartile concerning CLBP-related expenditures, exhibit an exponential pattern in utilizing healthcare services. These individuals demonstrate a mean cumulative cost of USD 31,459 within the initial five years post-diagnosis. Predominantly, these costs arise from outpatient services, encompassing therapeutic interventions, opioid pharmaceuticals, and spinal injections [8]. As such, the greatest opportunity to reduce HCU and lessen the disease burden may rely on addressing these high healthcare utilizers, which may have associated costly comorbidities such as diabetes, chronic opioid use, and pain that persists beyond two years [8].

CLBP remains the primary cause of work loss, the second most frequent reason to visit a healthcare professional, and the third most common indication for surgery [6,9]. A specific treatment for CLBP is recommended based on the underlying etiology and may range from pharmacological, non-pharmacological, interventional, and surgical options. Even after surgical treatment, up to 40% of patients may have persistent CLBP attributed to failed back surgery syndrome (FBSS) or post-laminectomy syndrome, with estimated healthcare costs of up to USD 20 billion [8,10].

Historically, spinal cord stimulation (SCS) has been used for the treatment of FBSS and complex regional pain syndrome (CRPS) [11,12]. Yet, recent advancements in technology and innovation in waveforms have yielded robust high-level clinical results, leading to the proposal for utilization of SCS earlier in the chronic pain treatment algorithm and for a broader range of painful conditions, such as painful diabetic neuropathy (PDN), chronic refractory neck pain, nonsurgical refractory back pain, in addition to FBSS and CRPS [13,14]. In particular, studies evaluating high-frequency (10 kHz) SCS for CLBP have demonstrated statistically significant and clinically meaningful improvement in pain and disability alone or when compared to conservative care [15,16,17,18,19,20,21,22].

The utilization of SCS therapy continues to grow as therapeutic indications expand, and it is estimated that nearly 50,000 SCS devices are implanted yearly in the United States, with an anticipated steady growth rate of up to 10% annually [23,24]. Similarly, the estimated increase in healthcare costs associated with CLBP, particularly in high utilizers, continues to generate a significant burden on individuals, caregivers, and the economy [25]. Therefore, given this simultaneous trend, this study aimed to analyze the impact of 10 kHz SCS therapy in HCU reduction, as measured by opioid utilization, number of outpatient visits for interventional pain procedures, and the number of emergency department (ED) visits related to the primary indication for 10 kHz SCS therapy.

## 2. Materials and Methods

### 2.1. Participants

The subjects of this study were adults at least 18 years of age with refractory to conservative care chronic pain of multiple etiologies, including postsurgical CLBP, CRPS, lumbar radiculopathy, PDN, and nonsurgical CLBP. Participants were included if they had a successful trial (>50% pain relief) with 10 kHz SCS therapy and subsequently underwent permanent percutaneous 10 kHz SCS implant with anatomical lead placement from 1 August 2019 to 31 December 2021. Selection of subjects was not restricted according to race, gender, socioeconomic status, healthcare insurance coverage, or any other demographic variable. Subjects were excluded if they did not meet the above criteria, if they had any absolute contraindication to percutaneous placement, such as uncontrolled coagulopathy, severe thrombocytopenia, active infection, or if they had been implanted with neuromodulation devices using waveforms other than 10 kHz. All participants included provided informed consent for the procedure and had at least 12 months (pre- and post-intervention) data for analysis.

### 2.2. Study Design and Data Collection

This was a retrospective single-center observational study, and Institutional Review Board (IRB) approval was obtained from The University of Kansas Medical Center IRB committee (IRB #00146998) prior to initiation. Data were collected from the institution’s electronic medical records database, and extraction was performed utilizing HERON software with support from the institution’s Clinical Informatics department. Data were cross checked for accuracy by the authors using the health system’s electronic health records and governmental prescription monitoring program online database. Importantly, there was no specific protocol by any of the physicians involved in this study to reduce opioid prescription prior to SCS implantation; therefore, subjects were included regardless of their opioid status at baseline and without a predefined tapering process. Using mean data for pre- and post-implant parameters and solving for 80% power, we would require only 41 pairs to detect statistically significant differences. This current study has 100 pairs; therefore, power is appropriate and almost 100%.

### 2.3. Outcome Measures

Demographic data extracted for analysis included age, gender, body mass index (BMI), presence of diabetes, psychiatric illness, smoking history, alcohol history, and history of spinal surgery. Outcome measures extracted for analysis were the number of outpatient visits for interventional treatment of pain (epidural steroid injections, transforaminal epidural steroid injections, lumbar radiofrequency ablation, medial branch bloc, sympathetic nerve blocks, joint injections, nerve blockers, etc.), morphine milligram equivalents (MME) to measure opioid dose utilization (limited to subjects taking opioids at baseline), and the number of emergency department visits associated with the primary diagnosis which SCS therapy was indicated.

### 2.4. Statistics

Data management and statistical analyses were performed using SAS software (version 9.4) (Copyright (c) 2002–2012 by SAS Institute Inc., Cary, NC, USA, All Rights Reserved). Categorical variables were summarized with percentages, and continuous variables were summarized by medians and means. Data were checked for normality. Comparisons between the pre- and post-10 kHz SCS implant responses were analyzed with Wilcoxon’s signed rank test for MME, ED visits, and outpatient visits. Comparisons between the low and high responders were analyzed with Wilcoxon Two-Sample Tests for self-reported pain improvement as well as MME, ED visits, and outpatient visits at both 12-month pre- and 12-month post-implant. Two-sided *p*-values less than 0.05 were considered statistically significant.

## 3. Results

The study population consisted of 160 subjects with a mean age of 62 years and a mean BMI of 32. Of these, 43% were males and 57% were females; 54% had no history of alcohol use, 62% had no history of tobacco use, and 68% had no history of diabetes. History of spinal surgery prior to 10 kHz SCS implant was present in 49% of participants, while 51% were participants with nonsurgical refractory back pain. Table 1 summarizes demographic and patient characteristics at baseline.

The overall self-reported improvement in pain among all participants was 67.5%. We further divided subjects into “high-responders” (≥80% self-reported pain relief) or “low-responders” (≥50% but ≤79% pain relief). This is the well-established cut off in the neuromodulation literature to delineate these subset cohorts [26,27,28,29]. Interestingly, 69.3% were self-reported “low responders” with a mean of 59.2% improvement in pain, while 30.6% were “high responders” with a mean of 86.4% self-reported improvement in pain. Baseline characteristics between ‘low’ and ‘high’ responder groups were statistically not significantly different.

Our study analyzed HCU trends among the overall cohort and these subset cohorts by measuring the mean number of ED visits, outpatient visits (interventional pain procedures), and opioid use by morphine milligram equivalent (MME) within 12 months prior and 12 months post-10 kHz SCS implant. All outcomes assessed demonstrated statistically significant reduction from pre- and post-10 kHz SCS implant follow-up. These findings are summarized in Table 2. There was no statistically significant difference between the subset cohorts. Table 3 summarizes their findings.

In our study, 62.5% of participants were taking opioids prior to 10 kHz SCS implant with a 51.0 MME and 26.5 MME average pre- and post-implant, respectively, representing a statistically significant (*p* < 0.0001) mean decrease of 24.5 MME overall and a mean of 78.2% dose reduction. Interestingly, 91.5% of these subjects reached the minimally clinically important difference (MCID) of a 30% decrease in MME from baseline that has been established in the literature [30]. Furthermore, within this cohort who decreased opioid use, 95.7% reduced their dose to <50 MME and 84.5% decreased to <30 MME within 12 months post-10 kHz SCS implant, while 37% completely discontinued opioid use. We tracked the frequency of ED visits per person and per year associated with the primary diagnosis up to 12 months prior and 12 months post-10 kHz SCS implant among our sample. We found a mean of 0.12 pre- and 0.03 post-implant, respectively, denoting a 0.08 reduction in ED visits pre- and post-10 kHz SCS implant, which was statistically significant (*p* < 0.01). Likewise, we assessed the rate number of outpatient visits per person and per year for interventional pain procedures related to the primary diagnosis up to 12 months prior and 12 months post-10 kHz SCS implant among our sample. We found a noteworthy decrease from a 1.39 mean pre- to 0.28 mean post-10 kHz SCS implant, representing a 1.11 mean reduction in outpatient visits, which was statistically significant (*p* < 0.0001).

## 4. Discussion

Our study found that 10 kHz SCS therapy significantly reduced opioid use, outpatient visits for interventional pain procedures, and emergency department visits up to 12-month follow-up post-10 kHz SCS implant. This is the first and only study evaluating HCU trends with 10 kHz SCS by assessing outpatient visits, ED visits, and opioid use collectively. Prior studies have analyzed these outcomes individually and demonstrated positive results with SCS therapy. Our study uniquely reports findings on one of the largest cohorts of real-world data published to date analyzing HCU trends with 10 kHz SCS for multiple pain etiologies, including post-laminectomy syndrome, CRPS, PDN, and nonsurgical refractory CLBP [31,32,33].

Within recent years, there has been an enduring effort to understand if SCS therapy may fit earlier in the treatment algorithm of CLBP by exploring HCU trends and cost-effectiveness. There are numerous high-quality level I-A and I-B studies to support SCS therapy with grade A recommendation with a strong level of certainty of substantial net benefit for CLBP following spinal surgery and grade B recommendation with a high level of certainty of moderate net benefit for nonsurgical CLBP [13]. Importantly, compared to conventional treatment for CLBP, SCS has been associated with favorable outcomes and found to be more cost-effective, with lower healthcare costs at 90 days [34,35,36,37].

### 4.1. Opioid Use

This single-center retrospective observational study revealed a statistically significant (*p* < 0.0001) decrease in opiate utilization with a mean of 78.2% dose reduction and 37% of participants completely discontinuing opioids. Remarkably, 91.5% of subjects reached MCID with at least a 30% decrease in dose from baseline [30]. There is vast literature reporting favorable results for opioid utilization with SCS therapy, independent of the waveform utilized [38]. Our findings reinforce the findings of previous studies reporting opioid utilization reduction with 10 kHz SCS therapy [39,40,41,42]. Kapural et al. analyzed patients with nonsurgical refractory back pain after a 10 kHz SCS implant at 12-month follow-up and reported a median reduction of 20 MME (*p*-value = 0.004), similar findings similar to ours with a mean reduction of 24.53 MME (*p*-value < 0.0001) [42]. Al-Kaisy et al. pooled data from two large prospective trials and performed a post-hoc analysis on opioid changes 12 months post-10 kHz SCS treatment. The authors found that 10 kHz SCS therapy reduced the overall dose of opioids and the proportion of subjects requiring high-risk doses >90 MME [39,40]. Similarly, Feng et al. reported a mean opioid dose reduction of 54%, while Gupta et al. described an 88% reduction or stable dose in opioids following a 10 kHz SCS implant [33,41].

According to prior studies, the delayed initiation of SCS therapy resulted in a 39% increase in odds of higher opioid utilization [34,43]. Patel et al. reported that the primary drivers for medication costs over a 6–12-month period were narcotics, with an average expenditure ranging from USD 131 to 519 per patient. The authors found a significant reduction in medication costs (*p* < 9.03) following 10 kHz SCS therapy [16]. Systematic and clinical reviews suggested that SCS may be associated with favorable results with opioid reduction or discontinuation [37,38,44]. Of note, the review by Al-Kaisy et al.’s review concluded that multiple prospective and retrospective studies demonstrated that 10 kHz SCS treatment reduced the mean dose of opioids and an average of over 60% of patients either reduced or eliminated opioids at the last follow-up [40]. Similarly, the review by Rupp et al.’s review concluded that SCS therapy has a positive impact on opioid reduction regardless of prior spinal surgery history; however, nonsurgical back subjects had the greatest mean opioid dose reduction of 50.39% MME and the greatest number of patients who discontinued opioids at 53.72% [38].

### 4.2. Emergency Department Service

Emergency department utilization by the chronic pain population is a useful indicator to gauge HCU [45]. Several studies have reported that individuals with CLBP have a markedly higher rate of ED utilization compared to the general population, with more than 4.3 million ED visits annually related to back pain [46]. Subjects with CLBP usually seek ED services due to limited access to primary care, lack of established care plans for their pain, and uncontrolled pain aiming for immediate pain relief [47]. Frequently, ED services are limited to imaging modalities to rule out urgent surgical needs and medication management and often lead to short-term opioids being utilized [48]. Interestingly, Figueroa et al. found certain factors that may predict a higher probability for CLBP subjects to seek ED services. These factors include younger age, lower income, near hospital housing, and opioid use at baseline; however, only opioid use was a significant predictor of seeking ED service in subjects with CLBP who underwent neuromodulation therapy [49].

In our study, we tracked HCU indicators, including opioid use reported above and the frequency of ED visits associated with the primary diagnosis up to 12 months pre- and post-10 kHz SCS implant. We found a statistically significant (*p* < 0.01) decrease in ED visits post-10 kHz SCS implant, with a mean of 0.12 pre- and 0.03 post-implant. Our findings align with previous studies supporting the longitudinal benefits of SCS to decrease ED visits as a marker of HCU reduction [50,51,52]. Similarly, Kapural et al. found a trend of decreased ED visits compared to baseline within 12 months post-10 kHz SCS, although this was not statistically significant. However, there was a statistically significant reduction in total outpatient office visits related to the primary diagnosis in subjects who underwent 10 kHz SCS [42].

### 4.3. Outpatient Visits for Interventional Pain Procedures

Our study did not examine outpatient office visits (primary care, specialty, or therapy) but rather tracked the number of outpatient interventional pain procedure visits in subjects who underwent 10 kHz SCS therapy as an indicator to measure HCU. We found a significant decrease from a 1.39 pre- to 0.28 post-mean after a 10 kHz SCS implant, representing a 1.11 mean reduction, which was statistically significant (*p* < 0.0001). These procedures included neuraxial injections, such as epidural steroid injections, transforaminal steroid injections, medial branch blocks, radiofrequency neurotomy, and sympathetic nerve blocks.

Our real-world data findings are largely in agreement with previous literature reporting statistically significant reductions in interventional pain procedures after neuromodulation therapy [33,53]. Kapural et al. found a statistically significant decrease in procedures from 2.2 ± 1.9 to 0.6 ± 1.2 in subjects with nonsurgical refractory CLBP, while Gupta et al. reported an 86% decline in the mean rate of interventional pain procedures for surgical and nonsurgical CLBP from 3.48 ± 3.05 per year prior to implantation to 0.49 ± 1.16 post 10 kHz SCS therapy [33,42]. Similarly, Di Benedetto et al. found a 72% reduction in interventional pain procedures in the neuromodulation group, compared to conservative care alone, while Patel et al. found similar statistically significant findings in interventional pain procedure reduction resulting in a 33% lower health care cost compared to conservative care [16,53]. Furthermore, Rajkumar et al. found a 59% HCU reduction after SCS implantation, the majority of which was for outpatient visits for interventional pain procedures [31,32].

### 4.4. Overall Health Care Cost Reduction

In this study, we report statistically significant HCU reduction after 10 kHz SCS in opioid use, ED visits, and outpatient visits for interventional pain procedures in a single-center retrospective analysis. Our findings should not be considered equivalent to a full cost-effective analysis, which was beyond the scope of this study. SCS therapy’s overall cost reduction and cost-effectiveness have been widely documented, and our findings align with these studies that expanded HCU trends to a more comprehensive exploration [36,54,55,56]. Systematic reviews concluded that high-frequency SCS therapy may be more cost-effective and cost-saving than conventional SCS and CMM for clinically approved indications [35,57]. Similar to our study, Gupta et al. reported single-center real-world data with diverse etiologies of CLBP and concluded that 10 kHz SCS therapy resulted in a weighted mean procedure cost reduction of USD 2528.74 per year per patient [33]. Patel et al. specifically evaluated nonsurgical CLBP subjects and found that 10 kHz SCS therapy resulted in a significant improvement in quality of life compared to CMM with lower cost based on HCU reduction and an ICER of USD 4964 at one year [16]. Taylor et al. found that 10 kHz SCS therapy resulted in lower rates of hospitalization and consequently lower health care costs among patients with PDN compared to CMM, with the CMM group’s total health care cost 51% higher, equivalent to a mean annual cost per patient of USD 9532 in the CMM and USD 6300 in the 10 kHz SCS group [58].

Delaying SCS therapy for approved indications is suboptimal and inversely proportional to the duration of diagnosis [59]. Longer pain-to-SCS time is associated with a significant increase in HCU and total medical expenditures. For every one-year increase in pain-to-SCS time, the odds increased by 33% for being in the high medical expenditures group, by 39% for being in the high opioid prescription group, and by 44% and 55% for being in the high office visits and hospitalizations group [43]. Importantly, the initial higher costs of SCS therapy can be counterbalanced by gains in work productivity, return to work, quality-adjusted life years (QALY), and enhancement in functional capacity [34]. These findings suggest that SCS might be a worthwhile therapy earlier in the treatment algorithm of approved clinical conditions [16,34].

Systematic reviews have evaluated numerous studies (randomized clinical trials, cost-utility analysis, pragmatic prospective studies) and concluded that there is moderate quality of evidence to support SCS therapy cost-effectiveness for approved clinical conditions [34,35,56]. SCS therapy is considered cost-effective compared to conventional medical management (CMM); however, this can depend on the indication, willingness-to-pay threshold, and time horizon analysis [34,35]. McClure et al.’s systematic review concluded that SCS provided both superior outcomes and a lower incremental cost-effectiveness ratio (ICER) compared to CMM and re-operation in patients with FBSS [56]. A key finding of this systematic review was that the break-even point where the difference in total costs was met by the savings per QALY was found at 24 months and beyond across the studies examined. Long-term cost savings suggested USD 6000 to 10,000 per QALY to insurers and national health services, when compared to CMM and re-operation with the overall medical cost of SCS found below USD 25,000/QALY [56]. Similar findings were reported by Odonkor et al.’s systematic review, which suggested that SCS therapy may provide higher incremental monetary value by decreasing long-term chronic pain burden at a lower ICER per QALY compared with CMM [34]. Although Dhruva et al. concluded no statistically significant cost-effectiveness between SCS therapy and CMM, the SCS group reported fewer interventional pain procedures at the first 12 months, yet not statistically significant at 24 months. Similarly, there was no difference between the two groups for hospitalizations or ED visits at two years of follow up [60]. However, one limitation of Dhruva et al. was follow-up time. Rajkumar et al. found that SCS therapy was associated with a significant decrease in total healthcare costs with a median total medical expenditure reduction of 59% after SCS implantation, concluding ultimately that the device covers the cost of acquisition at 27 months. These findings were based on large data sets containing claims data from commercial insurers, Medicaid and Medicare claims submitted by multiple health care providers in the United States [31,32]. Similarly, prior studies demonstrated cost-savings of approximately CAD 10,000 over a 5-year period and EUR 3513 in the United Kingdom with SCS therapy over CMM [61,62]. In agreement with these findings, Rojo et al. evaluated real-world data from the Spanish National Health Service and concluded that SCS therapy is cost-effective compared to CMM at five-year follow-up with 0.184 QALYs more for patients in the SCS group versus CMM [36]. Soreskog et al. corroborated such results by reporting an associated decrease of twenty-one disability days per patient and a decrease in indirect cost of EUR 4127 on sick leave and disability pension in patients with chronic neuropathic pain in a real-world population in Sweden population [63]. Finally, numerous other studies have shown that SCS is cost-effective in the long-term horizon across all insurance groups (Commercial, Medicaid, and Medicare) beginning at the 24-month mark and up to 9-year follow up [10,35,56,64].

### 4.5. Limitations

Our study has limitations. This is a non-blinded, non-randomized retrospective study without a control group, which could introduce the risk of selection bias. We tried to offset unintended bias by enrolling a large cohort of consecutive subjects with a broad eligibility criterion. We collected detailed HCU measures extracted from electronic health records within a single institution, yet given the nature of data extraction, some subjects may have sought care outside the institution within the follow-up period. Data extraction and verification were optimized by cross-checking with governmental prescription monitoring databases. Furthermore, the simplified approach involving a single-center retrospective analysis of the frequency of HCU events should not be considered equivalent to a full cost-effective analysis, which was beyond the scope of our study. Thereby, these factors may limit the generality and interpretation of results.

## 5. Conclusions

It is essential to determine the cost benefit of treatments for CLBP, given its continuous rise in HCU. Traditionally, SCS has been used for postsurgical CLBP and CRPS. However, with recent advancements in therapeutic indications, SCS utilization has expanded to include refractory nonsurgical CLBP and PDN treatment. Patients suffering from chronic pain are high utilizers of healthcare resources, particularly if pain persists beyond two years and if there are concomitant costly medical comorbidities. As such, the greatest opportunity to reduce HCU and lessen the disease burden may rely on addressing this population, for which the greatest drivers of medical expenditures are medication costs, outpatient services, and ED visits. Therefore, this study analyzed the impact of 10 kHz SCS therapy on these indicators of HCU.

This study found that 10 kHz SCS resulted in a statistically significant reduction of ED visits, outpatient visits for interventional pain procedures, and opioid use up to 12 months, with 91.5% of individuals reaching the MCID in opioid decline from baseline. This single-center retrospective observational study reports the largest cohort of real-world data published to date analyzing HCU trends with 10 kHz SCS for multiple pain etiologies, including postsurgical CLBP, CRPS, PDN, and nonsurgical refractory CLBP. This is the first and only study evaluating HCU trends with 10 kHz SCS by assessing outpatient visits, ED visits, and opioid use collectively. Prior studies have analyzed these outcomes individually and demonstrated positive results with SCS therapy similar to our findings.

## Figures and Tables

**Table 1:** Demographic and patient characteristics at baseline.

Characteristics at Baseline	Category	N(%) Total = 160
Gender	Male	69(43.1)
	Female	91(56.9)
History of Alcohol Use	No	87(54.4)
	Yes	73(45.6)
History of Tobacco Use	No	100(62.5)
	Yes	60(37.5)
History of Diabetes	No	110(68.8)
	Yes	50(31.3)
History of Psych Illness	No	73(45.6)
	Yes	87(54.4)
History of Spine Surgery	No	79(49.4)
	Yes	81(50.6)

**Table 2:** Healthcare utilization trend among the total cohort (N = 160).

Outcome	12-Month PRE-Implant Median/Mean +/− Std (95% CI)	12-Month POST-Implant Median/Mean +/− Std (95% CI)	Change	*p*-Value
Opioid use measured by MME	42/51.0 +/− 41.6 (42.80–59.32)	20/26.5 +/− 32.1 (20.14–32.90)	−24.5	<0.0001 *
ED visits	0/0.12 +/− 0.4 (0.05–0.18)	0/0.03 +/− 0.2 (0.00–0.06)	−0.09	0.01 *
Outpatient visits (interventional pain procedures)	1/1.39 +/− 1.8 (1.10–1.67)	0/0.28 +/− 0.6 (0.19–0.36)	−1.11	<0.0001 *

(*) Denotes statistically significant value; (−) denotes reduction in mean values. **Caption:** MME (morphine milligram equivalent); ED (emergency department).

**Table 3:** Outcome trend between low-responder and high-responder subset cohort.

Outcome Measurement	Low Responders 69.38% (N = 111) Median (Mean +/− Std)	High Responders 30.62% (N = 49) Median (Mean +/− Std)	*p*-Value
self-reported pain improvement	60 (59.23% +/− 8.9)	80 (86.43% +/− 7.8)	<0.0001 *
MME up to 12-month PRE-implant	40 (51.81 +/− 43.9)	45 (49.53 +/− 37.1)	1.00
MME up to 12-month POST-implant	20 (27.97 +/− 34.8)	20 (23.57 +/− 26)	0.75
ED visits up to 12-month PRE-implant	0 (0.12 +/− 0.4)	0 (0.12 +/− 0.3)	0.45
ED visits up to 12-month POST-implant	0 (0.04 +/− 0.2)	0 (0.02 +/− 0.1)	0.61
outpatient visits (interventional pain procedures) up to 12-month PRE-implant	1 (1.51 +/−1.9)	0 (1.10 +/− 1.7)	0.14
outpatient visits (interventional pain procedures) up to 12-month POST-implant	0 (0.29 +/− 0.6)	0 (0.24 +/− 0.5)	0.98

(*) Denotes statistically significant value. **Caption:** MME (morphine milligram equivalent); ED (emergency department).

## Data Availability

The data presented in this study are available on request from the corresponding author. The data are not publicly available due to privacy restrictions.

## References

[1] Dagenais S., Caro J., Haldeman S. (2008). A systematic review of low back pain cost of illness studies in the United States and internationally. Spine J..

[2] Andersson G.B. (1999). Epidemiological features of chronic low-back pain. Lancet.

[3] Wu A., March L., Zheng X., Huang J., Wang X., Zhao J., Blyth F.M., Smith E., Buchbinder R., Hoy D. (2020). Global low back pain prevalence and years lived with disability from 1990 to 2017: Estimates from the Global Burden of Disease Study 2017. Ann. Transl. Med..

[4] Spears C.A., Hodges S.E., Kiyani M., Yang Z., Edwards R.M., Musick A., Park C., Parente B., Lee H.J., Lad S.P. (2020). Health care resource utilization and management of chronic, refractory low back pain in the United States. Spine.

[5] Hartvigsen J., Hancock M.J., Kongsted A., Louw Q., Ferreira M.L., Genevay S., Hoy D., Karppinen J., Pransky G., Sieper J. (2018). What low back pain is and why we need to pay attention. Lancet.

[6] Vos T., Flaxman A.D., Naghavi M., Lozano R., Michaud C., Ezzati M., Shibuya K., Salomon J.A., Abdalla S., Aboyans V. (2012). Years lived with disability (YLDs) for 1160 sequelae of 289 diseases and injuries 1990-2010: A systematic analysis for the Global Burden of Disease Study 2010. Lancet.

[7] Ritzwoller D.P., Crounse L., Shetterly S., Rublee D. (2006). The association of comorbidities, utilization and costs for patients identified with low back pain. BMC Musculoskelet. Disord..

[8] Kirsch E.P., Yang L.Z., Lee H.J., Parente B., Lad S.P. (2023). Healthcare resource utilization for chronic low back pain among high-utilizers. Spine J..

[9] Patrick N., Emanski E., Knaub M.A. (2014). Acute and chronic low back pain. Med. Clin..

[10] Elsamadicy A.A., Farber S.H., Yang S., Hussaini S.M.Q., Murphy K.R., Sergesketter A., Suryadevara C.M., Pagadala P., Parente B., Xie J. (2017). Impact of Insurance Provider on Overall Costs in Failed Back Surgery Syndrome: A cost study of 122,827 patients. Neuromodulation.

[11] Lee A.W., Pilitsis J.G. (2006). Spinal cord stimulation: Indications and outcomes. Neurosurg. Focus.

[12] Sdrulla A.D., Guan Y., Raja S.N. (2018). Spinal cord stimulation: Clinical efficacy and potential mechanisms. Pain Pract..

[13] Sayed D., Grider J., Strand N., Hagedorn J.M., Falowski S., Lam C.M., Tieppo Francio V., Beall D.P., Tomycz N.D., Davanzo J.R. (2022). The American Society of Pain and Neuroscience (ASPN) evidence-based clinical guideline of interventional treatments for low back pain. J. Pain Res..

[14] Krog L., Maloney J., Pew S., Adeleye O., Johnson B., Glenn B., Gill B., Tieppo Francio V., Pagan-Rosado R., Whitney M. (2023). Cervical spinal cord stimulation: A review. Curr. Pain Headache Rep..

[15] Tieppo Francio V., Polston K.F., Murphy M.T., Hagedorn J.M., Sayed D. (2021). Management of chronic and neuropathic pain with 10 kHz spinal cord stimulation technology: Summary of findings from preclinical and clinical studies. Biomedicines.

[16] Patel N.P., Wu C., Lad S.P., Jameson J., Kosek P., Sayed D., Waldorff E.I., Shum L.C., Province-Azalde R., Kapural L. (2022). Cost-effectiveness of 10-kHz spinal cord stimulation therapy compared with conventional medical management over the first 12 months of therapy for patients with nonsurgical back pain: Randomized controlled trial. J. Neurosurg. Spine.

[17] Kapural L., Jameson J., Johnson C., Kloster D., Calodney A., Kosek P., Pilitsis J., Bendel M., Petersen E., Wu C. (2022). Treatment of nonsurgical refractory back pain with high-frequency spinal cord stimulation at 10 kHz: 12-month results of a pragmatic, multicenter, randomized controlled trial. J. Neurosurg. Spine.

[18] Billet B., Hanssens K., De Coster O., Santos A., Rotte A., Minne V. (2022). High-frequency (10 kHz) spinal cord stimulation for the treatment of focal, chronic postsurgical neuropathic pain: Results from a prospective study in Belgium. Pain Manag..

[19] Gupta M., Scowcroft J., Kloster D., Guirguis M., Carlson J., McJunkin T., Chaiban G., Israel A., Subbaroyan J. (2020). 10-kHz Spinal Cord Stimulation for Chronic Postsurgical Pain: Results from a 12-month prospective, multicenter study. Pain Pract..

[20] Kapural L., Yu C., Doust M.W., Gliner B.E., Vallejo R., Sitzman B.T., Amirdelfan K., Morgan D.M., Yearwood T.L., Bundschu R. (2016). Comparison of 10-kHz high-frequency and traditional low-frequency spinal cord stimulation for the treatment of chronic back and leg pain: 24-month results From a multicenter, randomized, controlled pivotal trial. Neurosurgery.

[21] Kapural L., Yu C., Doust M.W., Gliner B.E., Vallejo R., Sitzman B.T., Amirdelfan K., Morgan D.M., Brown L.L., Yearwood T.L. (2015). Novel 10-kHz high-frequency therapy (HF10 Therapy) is superior to traditional low-frequency spinal cord stimulation for the treatment of chronic Back and leg pain: The SENZA-RCT randomized controlled trial. Anesthesiology.

[22] Petersen E.A., Stauss T.G., Scowcroft J.A., Brooks E.S., White J.L., Sills S.M., Amirdelfan K., Guirguis M.N., Xu J., Yu C. (2021). Effect of high-frequency (10-kHz) spinal cord stimulation in patients with painful diabetic neuropathy: A randomized clinical trial. JAMA Neurol..

[23] Glenn B., Tieppo Francio V., Westerhaus B.D., Goree J., Strand N.H., Sparks D., Petersen E. (2023). Accessibility and ease of use in neuromodulation devices. Neuromodulation.

[24] Deer T.R., Pope J.E., Hayek S.M., Bux A., Buchser E., Eldabe S., De Andrés J.A., Erdek M., Patin D., Grider J.S. (2017). The Polyanalgesic Consensus Conference (PACC): Recommendations on intrathecal drug infusion systems best practices and guidelines. Neuromodulation.

[25] Fatoye F., Gebrye T., Ryan C.G., Useh U., Mbada C. (2023). Global and regional estimates of clinical and economic burden of low back pain in high-income countries: A systematic review and meta-analysis. Front. Public Health.

[26] Goudman L., Van Buyten J.P., De Smedt A., Smet I., Devos M., Jerjir A., Moens M. (2020). Predicting the response of high frequency spinal cord stimulation in patients with failed back surgery syndrome: A retrospective study with machine learning techniques. J. Clin. Med..

[27] Tieppo Francio V., Alm J., Leavitt L., Mok D., Yoon B.V., Nazir N., Lam C., Latif U., Sowder T., Braun E. (2023). Variables associated with nonresponders to high-frequency (10 kHz) spinal cord stimulation. Pain Pract..

[28] Duarte R.V., Soliday N., Leitner A., Taylor R.S. (2021). Health-related quality of life associated with pain health states in spinal cord stimulation for chronic neuropathic pain. Neuromodulation.

[29] Mekhail N., Levy R.M., Deer T.R., Kapural L., Li S., Amirdelfan K., Hunter C.W., Rosen S.M., Costandi S.J., Falowski S.M. (2020). Long-term safety and efficacy of closed-loop spinal cord stimulation to treat chronic back and leg pain (evoke): A double-blind, randomised, controlled trial. Lancet Neurol..

[30] Goudman L., Smedt A., Forget P., Moens M. (2020). Determining the minimal clinical important difference for medication quantification scale III and morphine milligram equivalents in patients with failed back surgery syndrome. J. Clin. Med..

[31] Rajkumar S., Yang L.Z., Venkatraman V., Charalambous L., Parente B., Lee H.J., Lad S.P. (2023). Health care resource utilization of high-frequency spinal cord stimulation for treatment of chronic refractory low back pain. Neuromodulation.

[32] Rajkumar S., Venkatraman V., Yang L.Z., Parente B., Lee H.J., Lad S.P. (2023). Short-term health care costs of high-frequency spinal cord stimulation for the treatment of postsurgical persistent spinal pain syndrome. Neuromodulation.

[33] Gupta M., Ray M., Ladesich N., Gupta A. (2021). Health-care utilization and outcomes with 10 kHz spinal cord stimulation for chronic refractory pain. J. Pain Res..

[34] Odonkor C.A., Orman S., Orhurhu V., Stone M.E., Ahmed S. (2019). Spinal cord stimulation vs. conventional therapies for the treatment of chronic low back and leg pain: A systematic review of health care resource utilization and outcomes in the last decade. Pain Med..

[35] Niyomsri S., Duarte R.V., Eldabe S., Fiore G., Kopell B.H., McNicol E., Taylor R.S. (2020). A systematic review of economic evaluations reporting the cost-effectiveness of spinal cord stimulation. Value Health.

[36] Rojo E., Pérez Hernández C., Sánchez Martínez N., Margarit A.C., Blanco Arias T., Muñoz Martínez M., Crespo C., Ochoa Mazarro D. (2021). Real-world cost-effectiveness analysis of spinal cord stimulation vs. conventional therapy in the management of failed back surgery syndrome. J. Pain Res..

[37] Duarte R.V., Bentley A., Soliday N., Leitner A., Gulve A., Staats P.S., Sayed D., Falowski S.M., Hunter C.W., Taylor R.S. (2023). Cost-utility analysis of evoke closed-loop spinal cord stimulation for chronic back and leg pain. Clin. J. Pain.

[38] Rupp A., Francio V.T., Hagedorn J.M., Deer T., Sayed D. (2022). The impact of spinal cord stimulation on opioid utilization in failed back surgery syndrome and surgery naive patients. Interv. Pain Med..

[39] Al-Kaisy A., Van Buyten J.P., Carganillo R., Caraway D., Gliner B., Subbaroyan J., Panwar C., Rotte A., Amirdelfan K., Kapural L. (2019). 10 kHz SCS therapy for chronic pain, effects on opioid usage: Post hoc analysis of data from two prospective studies. Sci. Rep..

[40] Al-Kaisy A., Van Buyten J.P., Amirdelfan K., Gliner B., Caraway D., Subbaroyan J., Rotte A., Kapural L. (2020). Opioid-sparing effects of 10 kHz spinal cord stimulation: A review of clinical evidence. Ann. N. Y. Acad. Sci..

[41] Feng H., Doherty P., Rotte A. (2021). Decreased opioid consumption and durable pain relief in patients treated with 10 kHz SCS: A retrospective analysis of outcomes from single-center. J. Pain Res..

[42] Kapural L., Calodney A. (2022). Retrospective efficacy and cost-containment assessment of 10 kHz Spinal Cord Stimulation (SCS) in non-surgical refractory back pain patients. J. Pain Res..

[43] Lad S.P., Iii F.W.P., Kent A.R., Cook S., Murphy K.R., Dalal N., Karst E., Staats P., Sharan A. (2016). Longer delay from chronic pain to spinal cord stimulation results in higher healthcare resource utilization. Neuromodulation.

[44] Vu T.N., Khunsriraksakul C., Vorobeychik Y., Liu A., Sauteraud R., Shenoy G., Liu D.J., Cohen S.P. (2022). Association of spinal cord stimulator implantation with persistent opioid use in patients with postlaminectomy syndrome. JAMA Netw. Open.

[45] Depelteau A., Racine-Hemmings F., Lagueux E., Hudon C. (2020). Chronic pain and frequency use of emergency department: A systematic review. Am. J. Emerg. Med..

[46] Magel J., Suslavich K., Roper K., Fritz J., Madsen T. (2022). Emergency department evaluation, treatment, and functional outcomes among patients presenting with low back pain. Am. J. Emerg. Med..

[47] Jambunathan J., Chappy S., Siebers J.J., Deda A. (2016). Patient-centered care for chronic pain in the emergency department: A qualitative study. Int. Emerg. Nurs..

[48] Abdolrazaghnejad A., Banaie M., Tavakoli N., Safdari M., Rajabpour-Sanati A. (2018). Pain management in the emergency department: A review articule on options and methods. Adv. J. Emerg. Med..

[49] Figueroa C., Hadanny A., Kroll K., DiMarzio M., Ahktar K., Gillogly M., Mitchell D., Cangero T., Pilitsis J.G. (2021). Does neuromodulation reduce chronic pain patient emergency department utilization?. Neurosurgery.

[50] Kumar K., Taylor R.S., Jacques L., Eldabe S., Meglio M., Molet J., Thomson S., O’Callaghan J., Eisenberg E., Milbouw G. (2007). Spinal cord stimulation versus conventional medical management for neuropathic pain: A multicentre randomised controlled trial in subjects with failed back surgery syndrome. Pain.

[51] Zucco F., Ciampichini R., Lavano A., Costantini A., De Rose M., Poli P., Fortini G., Demartini L., De Simone E., Menardo V. (2015). Cost-effectiveness and cost-utility analysis of spinal cord stimulation in subjects with failed back surgery syndrome: Results from the PRECISE study. Neuromodulation.

[52] Mekhail N.A., Aeschbach A., Stanton-Hicks M. (2004). Cost benefit analysis of neurostimulation for chronic pain. Clin. J. Pain.

[53] Di Benedetto D.J., Wawrzyniak K.M., Schatman M.E., Kulich R.J., Finkelman M. (2018). 10 kHz spinal cord stimulation: A retrospective analysis of real-world data from a community-based, interdisciplinary pain facility. J. Pain Res..

[54] Taylor R.S., Ryan J., O’Donnell R., Eldabe S., Kumar K., North R.B. (2010). The cost-effectiveness of spinal cord stimulation in the treatment of failed back surgery syndrome. Clin. J. Pain.

[55] Kumar K., Rizvi S. (2013). Cost-effectiveness of spinal cord stimulation therapy in management of chronic pain. Pain Med..

[56] McClure J.J., Desai B.D., Ampie L., You W., Smith J.S., Buchholz A.L. (2021). A systematic review of the cost-utility of spinal cord stimulation for persistent low back pain in patients with failed back surgery syndrome. Glob. Spine J..

[57] Taylor R.S., Bentley A., Campbell B., Murphy K. (2020). High-frequency 10 kHz spinal cord stimulation for chronic low back and leg pain: Cost-consequence and cost-effectiveness analyses. Clin. J. Pain.

[58] Taylor R.S., Lad S.P., White J.L., Stauss T.G., Healey B.E., Sacks N.C., McLin R., Patil S., Jaasma M.J., Caraway D.L. (2023). Health care resource utilization and costs in patients with painful diabetic neuropathy treated with 10kHz spinal cord stimulation therapy. J. Manag. Care Spec. Pharm..

[59] Kumar K., Rizvi S., Nguyen R., Abbas M., Bishop S., Murthy V. (2014). Impact of wait times on spinal cord stimulation therapy outcomes. Pain Pract..

[60] Dhruva S.S., Murillo J., Ameli O., Morin P.E., Spencer D.L., Redberg R.F., Cohen K. (2023). Long-term outcomes in use of opioids, nonpharmacologic pain interventions, and total costs of spinal cord stimulators compared with conventional medical therapy for chronic pain. JAMA Neurol..

[61] Mekhail N., Wentzel D.L., Freeman R., Quadri H. (2011). Counting the costs: Case management implications of spinal cord stimulation treatment for failed back surgery syndrome. Prof. Case Manag..

[62] Annemans L., Van Buyten J.P., Smith T., Al-Kaisy A. (2014). Cost effectiveness of a novel 10 kHz high-frequency spinal cord stimulation system in patients with failed back surgery syndrome (FBSS). J. Long Term Eff. Med. Implant..

[63] Söreskog E., Jacobson T., Kirketeig T., Fritzell P., Karlsten R., Zethraeus N., Borgström F. (2023). Impact of spinal cord stimulation on sick leave and disability pension in patients with chronic neuropathic pain: A real-world evidence study in Sweden. Pain.

[64] Farber S.H., Han J.L., Elsamadicy A.A., Hussaini Q., Yang S., Pagadala P., Parente B., Xie J., Lad S.P. (2017). Long-term cost utility of spinal cord stimulation in patients with failed back surgery syndrome. Pain Physician.

